# WaveST-Yield: a novel spatio-temporal deep learning framework with frequency-domain refinement for UAV-based maize yield prediction

**DOI:** 10.3389/fpls.2026.1838598

**Published:** 2026-05-29

**Authors:** Huiqin Li, Pengzhi Hou, Runqing Zhang, Xindan Zhang, Jinfeng Peng, Xiao Cui, Fuzhong Li, Xiaoying Zhang

**Affiliations:** 1 Faculty of Software Technologies, Shanxi Agricultural University, Jinzhong, China; 2 School of Mathematical and Computational Sciences, College of Sciences, Massey University, Auckland, New Zealand

**Keywords:** attention mechanism, deep learning, maize yield prediction, precision agriculture, spatio-temporal modeling, UAV multispectral remote sensing, wavelet transform

## Abstract

Accurate and generalizable plot-scale maize yield prediction is critical for precision agriculture and food security. While UAV-based multispectral remote sensing provides rich phenotyping data, existing yield prediction models often struggle with insufficient mining of complex spatio-temporal dynamics, ineffective separation of spatial details from background noise, and inadequate focus on yield-sensitive features throughout the crop growth cycle. To address these limitations, this study proposes WaveST-Yield, a novel hybrid deep learning framework tailored for multi-temporal multispectral data. The proposed model integrates three core modules: a Spatio-Temporal Phenology Encoder (SPE) based on ConvLSTM to capture the temporal dynamic patterns and spatio-temporal correlations across the entire growth period; a Multiscale Frequency-Spatial Refiner (MFSR) utilizing Haar Wavelet Downsampling (HWD) to preserve image details and decouple noise without early loss of key physiological features; and an Adaptive Yield-Sensitive Re-calibrator (AYSR) leveraging a 3D-CBAM attention mechanism to enhance the extraction of critical yield-related traits while suppressing background interference. The model was rigorously evaluated on two independent maize experimental fields using 5-fold cross-validation and cross-plot external validation. Results demonstrate that WaveST-Yield consistently outperforms traditional machine learning algorithms and single-structure deep learning models, achieving the highest prediction accuracy (Overall R² of 0.883 and 0.775 in Field 1 and Field 2, respectively) with superior error control. Extensive ablation and multi-model comparison experiments confirm that the synergistic integration of spatio-temporal encoding, frequency-domain refinement, and 3D attention mechanisms significantly improves model robustness and cross-regional generalization ability. This study provides a highly accurate, robust, and generalizable methodological framework for high-throughput crop yield monitoring.

## 1 Introduction

Accurate and generalizable prediction of plot-scale maize yield is critical for optimizing agronomic management, shortening breeding cycles, and safeguarding regional food security (Hove et al., 2025). Maize is a core staple cereal crop worldwide that ensures grain supply and supports the development of the livestock and grain processing industries (Wu and Guclu, 2013). Accurate prediction of maize yield has irreplaceable strategic value for the optimization of crop cultivation management, evaluation of variety breeding, and early warning of regional food security (Stevanović et al., 2025). Traditional maize yield acquisition relies on field-based manual yield measurement, which has inherent limitations including being time-consuming and labor-intensive, requiring destructive sampling, and having poor spatial representativeness. These drawbacks make it difficult to meet the core demands of modern breeding and smart agriculture for plot-scale, high-throughput, and non-destructive yield monitoring (Radočaj et al., 2025). With the rapid development of remote sensing technology, UAV-borne multi-spectral sensors have become a mainstream technical approach for crop phenotyping analysis and yield prediction, owing to their advantages of high spatiotemporal resolution, flexible operation, and low cost (Zhang et al., 2025). How to achieve accurate, robust, and generalizable maize yield prediction based on UAV multi-temporal multi-spectral data has become a prominent research hotspot and key scientific issue in the fields of agricultural remote sensing and smart farming.

UAV-based multi-temporal multi-spectral remote sensing can continuously acquire the spectral reflection characteristics, spatial texture heterogeneity, and temporal growth dynamics of maize canopy throughout the whole growth period, providing a rich data source for mining the inherent correlation between phenotypic traits and yield formation (Yang et al., 2025). At present, most existing studies still take single or a small number of vegetation indices, such as the Normalized Difference Vegetation Index (NDVI) and Green Normalized Difference Vegetation Index (GNDVI), as the core inputs, and construct yield prediction models through methods including linear regression and machine learning. Guo et al. (2024) proposed the Multi-Spectral Composite Vegetation Index (MSCVI) integrating UAV RGB-based volume index and multi-spectral vegetation indices, and combined it with Back Propagation (BP) neural network and Random Forest (RF) models to predict the yield of irrigated maize over three years, verifying the effectiveness of the index and the robustness of the models. Bao et al. (2024) used UAV-acquired canopy vegetation indices and crop phenological metrics, combined with three models including Ordinary Least Squares (OLS), Step-wise Multiple Linear Regression (SMLR), and Gradient Boosting Regression Tree (GBRT), to predict spring maize yield. They verified the feasibility of yield prediction using UAV imagery, and found that the grain-filling stage was the optimal prediction period and the GBRT model achieved the best prediction performance. Kumar et al. (2023) adopted a method combining VIs derived from UAV multi-spectral data with five machine learning models, namely Linear Regression (LR), k-Nearest Neighbor (KNN), Random Forest (RF), Support Vector Regression (SVR), and Deep Neural Network (DNN) at the farm scale. They conducted yield prediction research using limited training samples at the maize vegetative growth stage (V6) and reproductive growth stage (R5), and evaluated the effects of four agronomic treatments on maize yield. Such methods are highly dependent on handcrafted features, making it difficult to fully mine the implicit spatial structure and temporal variation information contained in the imagery, and have poor adaptability to the environmental heterogeneity across different plots.

Furthermore, a single band or vegetation index is difficult to fully characterize the physiological growth status of the maize canopy. The effective utilization of multi-band and multi-vegetation index data, as well as the construction of plot-scale standardized data cubes, remain key technical bottlenecks for improving the accuracy and generalization ability of remote sensing-based yield prediction. Yan et al. (2026) integrated UAV four-band vegetation indices and meteorological data in the Lifang Dryland Experimental Area of Shanxi Province, and adopted eight machine learning algorithms to conduct maize yield prediction across multiple growth periods. They identified key features and their spatial distribution through SHapley Additive exPlanations (SHAP) analysis and spatial clustering, and found that the Random Forest (RF) and Gradient Boosting Tree (GBT) models achieved the best performance, with a maximum R² of 0.8696 and a relative Root Mean Square Error (rRMSE) of approximately 2.8%, providing a quantitative framework for spatial management in arid regions. Bao et al. (2025) combined UAV-derived vegetation indices and maize agronomic traits at Youyi Farm in Heilongjiang Province, and constructed yield prediction models for 18 planting treatments using RF. The model achieved the optimal accuracy (R²=0.74) after integrating the Normalized Difference Red Edge (NDRE), Leaf Area Index (LAI), and Soil and Plant Analyzer Development (SPAD) values of middle-layer leaves at the milk-ripening stage, providing technical support for field precision agriculture. Wang et al. (2025) combined 13 UAV-derived vegetation indices with maize phenological periods in the Loess Plateau, and constructed yield inversion models for different plastic film mulching and nitrogen application treatments. The Enhanced Vegetation Index 2 (EVI2) showed the strongest correlation with LAI, and the best prediction performance was obtained at the tasseling stage, with a maximum R² of 0.94, providing empirical support for precision agriculture. All three studies relied on traditional machine learning algorithms, failed to fully mine the deep nonlinear correlation between remote sensing and agronomic data, and the generalization ability of the models was significantly limited by regional environments and planting conditions.

The rise of deep learning technology has provided a novel solution for the end-to-end deep mining of agricultural remote sensing data. Among them, the 3D-CNN can synchronously extract joint spatial-spectral features, and the ConvLSTM network can model temporal dynamics while preserving the spatial structure of input data. Both have shown significantly better performance than traditional machine learning in the field of crop phenotypic prediction (Li et al., 2020; Nejad et al., 2024);. Wang et al. (2023) proposed a novel method combining dimensionality reduction approaches with 3D-CNN, and introduced metric learning and multi-task learning into the framework. This method makes full use of the spatiotemporal features of multi-source spatial images, addresses the limitation that existing crop yield prediction methods fail to fully mine the spatiotemporal information and dimensionality reduction potential of the data, and has been verified to be effective in county-level soybean yield prediction in the United States, with improved yield estimation accuracy. Tian et al. (2021) constructed a wheat yield estimation model in the Guanzhong Plain by fusing meteorological data and remote sensing indices including the Vegetation Temperature Condition Index (VTCI) and LAI using a LSTM network. The model with dual time-step combined input achieved the optimal accuracy (R²=0.83), which was significantly better than the Back Propagation Neural Network (BPNN) and Support Vector Machine (SVM), and had good adaptability under different irrigation districts and climatic conditions. However, the former used 3D-CNN alone and the latter used ConvLSTM alone, lacking the organic integration of temporal sequence encoding and spatiotemporal feature extraction. These models are prone to overfitting and insufficient generalization in agricultural plot scenarios with small sample sizes and high heterogeneity. Muruganantham et al. (2022) systematically reviewed the literature from 2012 to 2022, summarized the application of deep learning and remote sensing technology in crop yield prediction, pointed out that LSTM and CNN are the most commonly used deep learning methods, while satellite remote sensing and vegetation indices are the mainstream data sources and input features. Meanwhile, they also mentioned core challenges including accuracy improvement, model practical application, and the black-box problem of deep learning models. In addition, there is still a lack of systematic and standardized methodological system support for deep learning architecture design, hyperparameter optimization strategy, and cross-plot generalization validation targeting multi-temporal multispectral data of maize throughout the whole growth period. Jabed and Murad (2024) addressed the low accuracy of traditional machine learning in crop yield prediction, systematically reviewed the progress of machine learning and deep learning in this field, summarized mainstream models for diverse crops, and put forward future research suggestions.

To address the issues in current UAV-based multispectral maize yield prediction, including insufficient mining of multispectral temporal features, difficulty in effectively separating original image details from noise, and insufficient response of the model to yield-sensitive features, which result in prediction accuracy failing to meet the requirements of practical applications, this paper proposes WaveST-Yield, a hybrid model composed of three core modules: the Spatio-Temporal Phenology Encoder (SPE) based on the ConvLSTM architecture, which can fully capture the temporal dynamic patterns and spatio-temporal correlation characteristics of multispectral data throughout the maize growth cycle; the Multiscale Frequency-Spatial Refiner (Xu et al., 2023), which replaces the first-layer downsampling of the conventional 3D-CNN with Haar Wavelet Downsampling (HWD) to achieve effective fidelity preservation of multispectral image details and noise decoupling, thereby avoiding the loss of key physiological features in the initial stage of the network; and the Adaptive Yield-Sensitive Re-calibrator (AYSR) based on CBAM (Woo et al., 2018), which embeds the 3D-CBAM attention mechanism after feature extraction to enhance the model’s ability to extract yield-sensitive features while suppressing interference from irrelevant background information. To avoid model overfitting and the randomness of experimental results, and to ensure the reliability of prediction performance evaluation, this study adopts five-fold cross-validation for model training and testing; meanwhile, cross-plot validation is used to verify the generalization ability of the model, and comparative experiments are carried out with a variety of mainstream prediction models. The experimental results show that the proposed model achieves the optimal performance in both prediction accuracy and generalization ability.

## 2 Materials and methods

### 2.1 Materials

#### 2.1.1 Experimental field and plot layout

**Figure 1:**
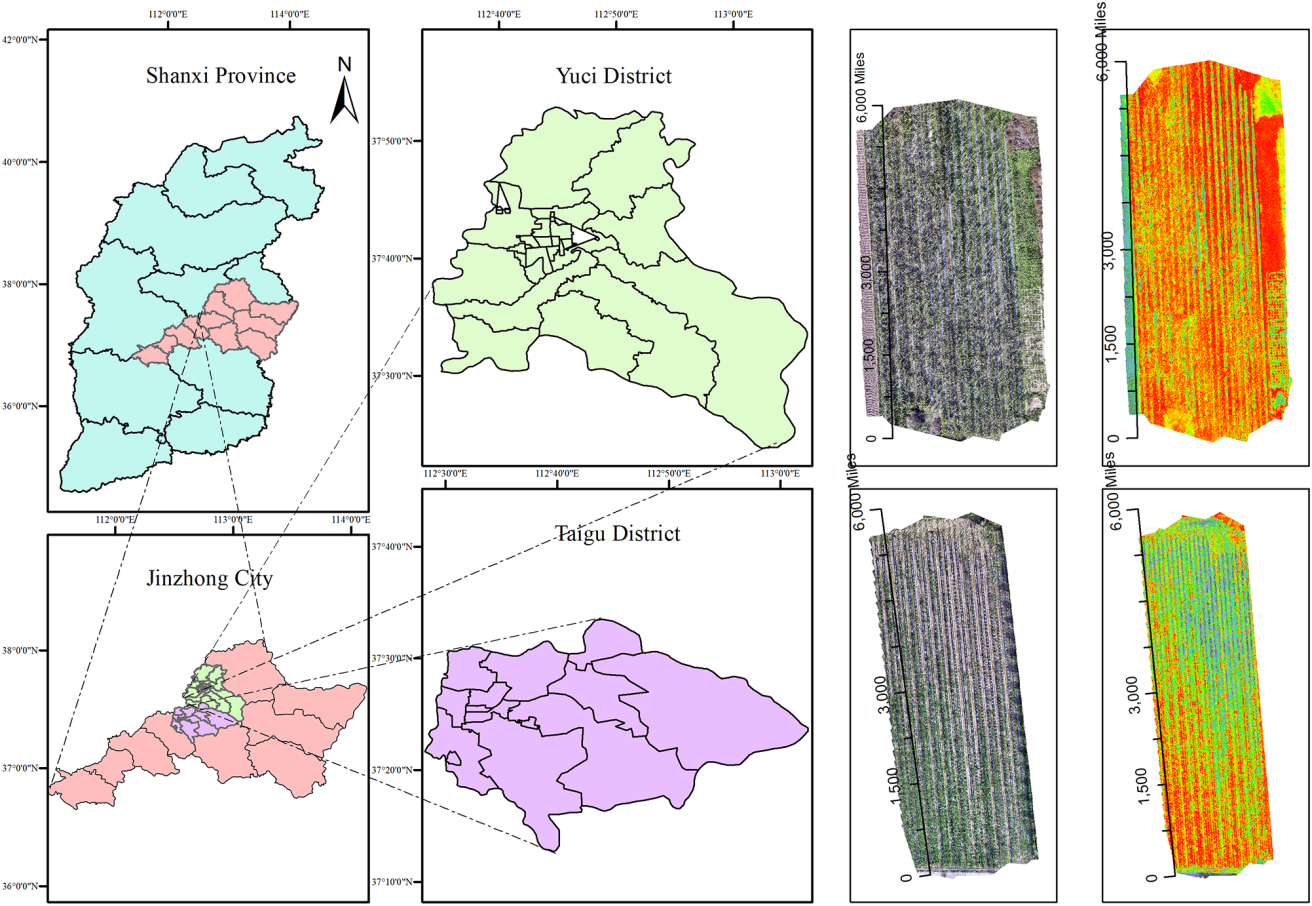
Location of the study area and UAV multispectral orthomosaics of the experimental fields.

In 2025, we established two independent maize experimental fields in Dongyang Town, Yuci District, Jinzhong City, Shanxi Province, China (37.55°N, 112.67°E) and Taigu District, Jinzhong City, Shanxi Province, China (37.41°N, 112.59°E), as shown in **Figure 1**. Both fields had flat terrain with consistent irrigation regimes and field management practices, making them suitable for plot-scale multi-temporal phenotyping monitoring and yield modeling.

To characterize the soil background conditions of the experimental sites, we determined the physicochemical properties of the soil in the plots. The soil was overall slightly alkaline, with a pH of 8.46 ± 0.07, soil organic matter of 19.62 ± 1.72 g·kg^−1^, available phosphorus of 14.70 ± 1.23 mg·kg^−1^, available potassium of 175.56 ± 16.58 mg·kg^−1^, nitrate nitrogen (NO_3_^−^-N) of 5.93 ± 0.45 mg·kg^−1^, and ammonium nitrogen (NH_4_^+^-N) of 13.83 ± 1.34 mg·kg^−1^.

A single summer maize cultivar, Shuanghui 207, was planted in both experimental fields, with sowing and transplanting completed on August 1. The crop was established via greenhouse seedling raising followed by mechanical transplanting and field planting using a seedling planter, to minimize non-experimental errors among plots caused by uneven seedling emergence. The fields were arranged in a regular matrix design, with a total of 161 plots in each experimental field. Each individual plot covered an area of approximately 4 m², with a row spacing of 60 cm and a plant spacing of 30 cm. To ensure the comparability and traceability of plot-scale phenotypic information, the vector boundary of each individual plot was adopted as the sole spatial analysis unit in this study. All subsequent multi-spectral image cropping, feature extraction, and model input data preparation were strictly conducted within these boundaries.

#### 2.1.2 Measured response variable

**Figure 2:**
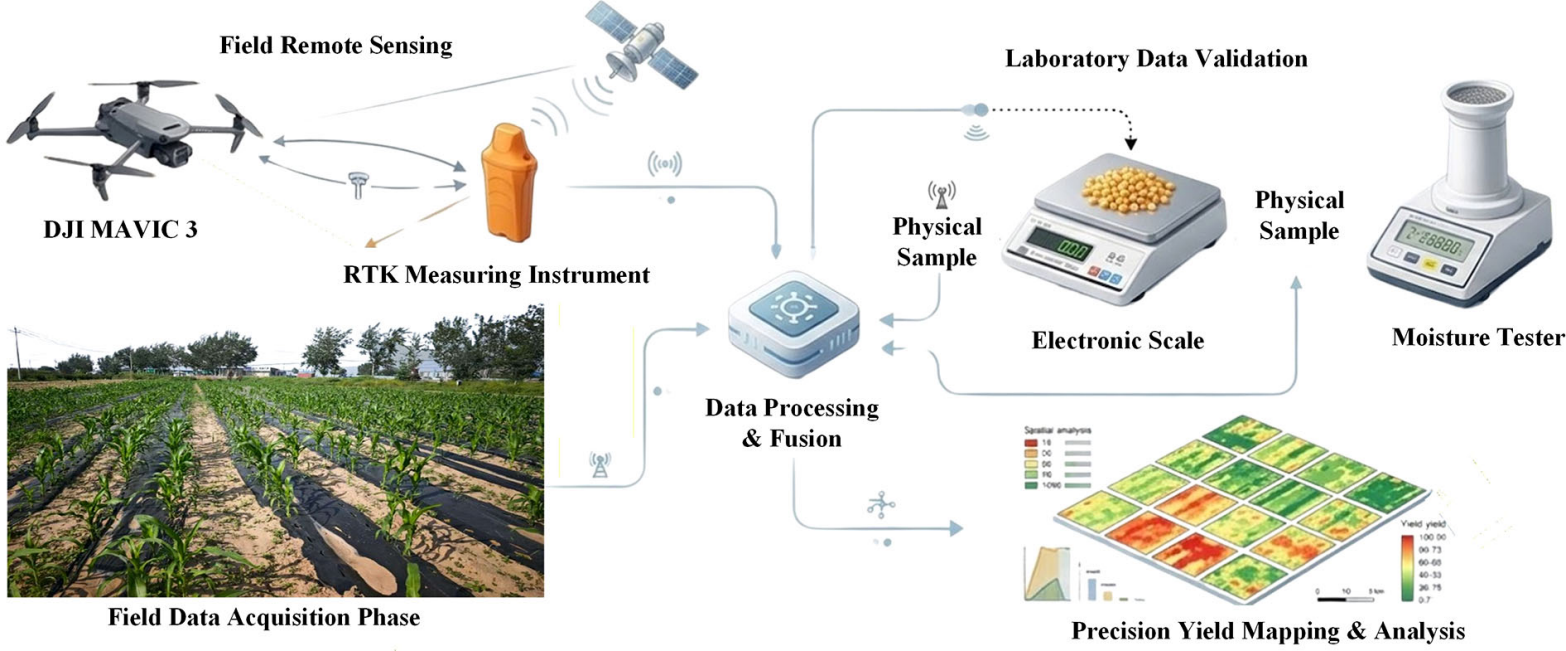
Data collection and processing workflow, including UAV flight, RTK measurement, laboratory grain processing, and moisture content determination.

At the physiological maturity and harvest stage of maize, independent harvesting and yield measurement were carried out on a per-plot basis. After harvesting maize ears from each plot, manual threshing and impurity removal were performed to obtain grain samples, as shown in **Figure 2**. The fresh weight of the grains was weighed and recorded using an electronic balance. Subsequently, the grain moisture content was determined with a grain moisture analyzer, KAITE model PM-8188-A. Triplicate measurements were conducted for each plot, and the mean value was adopted. To eliminate the interference of differences in harvested grain moisture content among plots on yield comparison, the grain weight was uniformly converted to the standard moisture content of 14% (Killeen et al., 2024), and further converted to yield per unit area (t·ha^−1^) according to the plot area, which was taken as the response variable y of the model.

#### 2.1.3 Multi-spectral data acquisition and processing for UAVs

Multi-spectral imagery was acquired using a DJI Mavic 3 UAV platform, equipped with a UAV-borne multi-spectral camera featuring 4 narrow bands: Green (G), Red (R), Red Edge (RE), Near-Infrared (NIR) and 5 megapixels (MP) per channel, as well as a 20 MP RGB camera for visible light recording.

Aerial surveys were conducted in stages at key growth periods of maize, with a total of 12 phases of imagery acquired for Field 1 and 9 phases of imagery for Field 2. All flight missions were performed with pre-programmed autonomous flight routes at an altitude of approximately 30 m above ground level. Flights were preferably carried out between 10:00 and 14:00 local time under clear sky conditions with stable solar irradiance. The camera pitch angle was set to a near-nadir view, approximately −70° to −80°, to minimize the impacts of shadowing and viewing geometry variations on the consistency of spectral reflectance.

The raw imagery was processed via DJI Terra 4.8.0 to complete radiometric calibration, geometric correction, and orthomosaic, generating reflectance orthomosaics with a spatial resolution of 0.05 m. Field plot boundary acquisition was conducted using a Stonex Orange M10 inertial navigation system (INS) integrated RTK surveying instrument, as shown in **Figure 2**. Relying on full-constellation GNSS and integrated positioning with built-in inertial navigation, and cooperating with a base station to achieve tilt measurement and engineering-grade centimeter-level positioning, the geographic coordinates under the WGS84 coordinate system were collected via SurPad 4.2 software to ensure a unified spatial reference.

Spatial registration between the plot vector boundaries and the orthomosaics was completed in ArcMap 10.6.1. Buffers were created centered on the sampling points, and after topology checks, the imagery was cropped into rectangular image tiles of 2.0m × 2.0m. Based on Python 3.8.0 combined with the GDAL and NumPy libraries, a plot-level three-dimensional spatiotemporal-spectral data cube was constructed sequentially over time.

Subsequently, a multi-temporal data cube was constructed for each plot in chronological order using Python 3.8.0, denoted as {X|X∈RN×T×H×W×C}, where N is the number of plot samples, T is the number of temporal phases (Field 1: 12; Field 2: 9), H×W is the spatial dimension of the cropped plot image tiles, and C = 4 is the number of multi-spectral bands. To eliminate inconsistencies in cropped dimensions among plots, bilinear interpolation was adopted in the data loading stage to uniformly resample all samples to (H′, W′)=(160, 160).

Based on the four-band reflectance, this study further calculated 15 commonly used VIs, which were concatenated with the original bands along the channel dimension to obtain a 19-channel input (4 bands + 15 VIs). Therefore, the final input of the deep learning model is denoted as {X~|X~∈RN×T×H′×W′×19}. To adapt to the deep learning framework, the input tensor was formatted in channel−first order as (T, C, H′, W′). The definitions and formulas of each band and vegetation index are shown in **Table 1**.

**Table 1:** Definition and calculation formulas of each spectral band and vegetation index.

SN	Type	Name of factors	Wavelength/equation
1	Band	Green band reflectance (G)	560 ± 16 nm
2	Band	Red band reflectance (R)	650 ± 16 nm
3	Band	Red-edge band reflectance (RE)	730 ± 16 nm
4	Band	Near-infrared band reflectance (NIR)	860 ± 26 nm
5	VI	Normalized Difference Vegetation Index (NDVI)	(NIR − R)/(NIR + R)
6	VI	Green Normalized Difference Vegetation Index (GNDVI)	(NIR − G)/(NIR + G)
7	VI	Normalized Difference Red Edge Index (NDRE)	(NIR − RE)/(NIR + RE)
8	VI	Soil Adjusted Vegetation Index (L = 0.5) (SAVI)	(1+L)·(NIR − R)/(NIR + R + L), L = 0.5
9	VI	Modified Soil Adjusted Vegetation Index (MSAVI)	(2·NIR + 1 − √((2·NIR + 1)^2 − 8·(NIR − R)))/2
10	VI	Optimized Soil Adjusted Vegetation Index (OSAVI)	(NIR − R)/(NIR + R + 0.16)
11	VI	Enhanced Vegetation Index 2 (EVI2)	2.5·(NIR − R)/(NIR + 2.4·R + 1)
12	VI	Difference Vegetation Index (DVI)	NIR − R
13	VI	Ratio Vegetation Index/Simple Ratio (RVI (SR))	NIR/R
14	VI	Renormalized Difference Vegetation Index (RDVI)	(NIR − R)/√(NIR + R)
15	VI	Modified Simple Ratio (MSR)	(NIR/R − 1)/√(NIR/R + 1)
16	VI	Chlorophyll Index (Green) (CIgreen)	NIR/G − 1
17	VI	Chlorophyll Index (Red Edge) (CIre)	NIR/RE − 1
18	VI	Normalized Green–Red Difference Index (NGRDI)	(G − R)/(G + R)
19	VI	Triangular Vegetation Index (TVI)	0.5·[120·(NIR − G) − 200·(R − G)]

### 2.2 Methods

**Figure 3:**
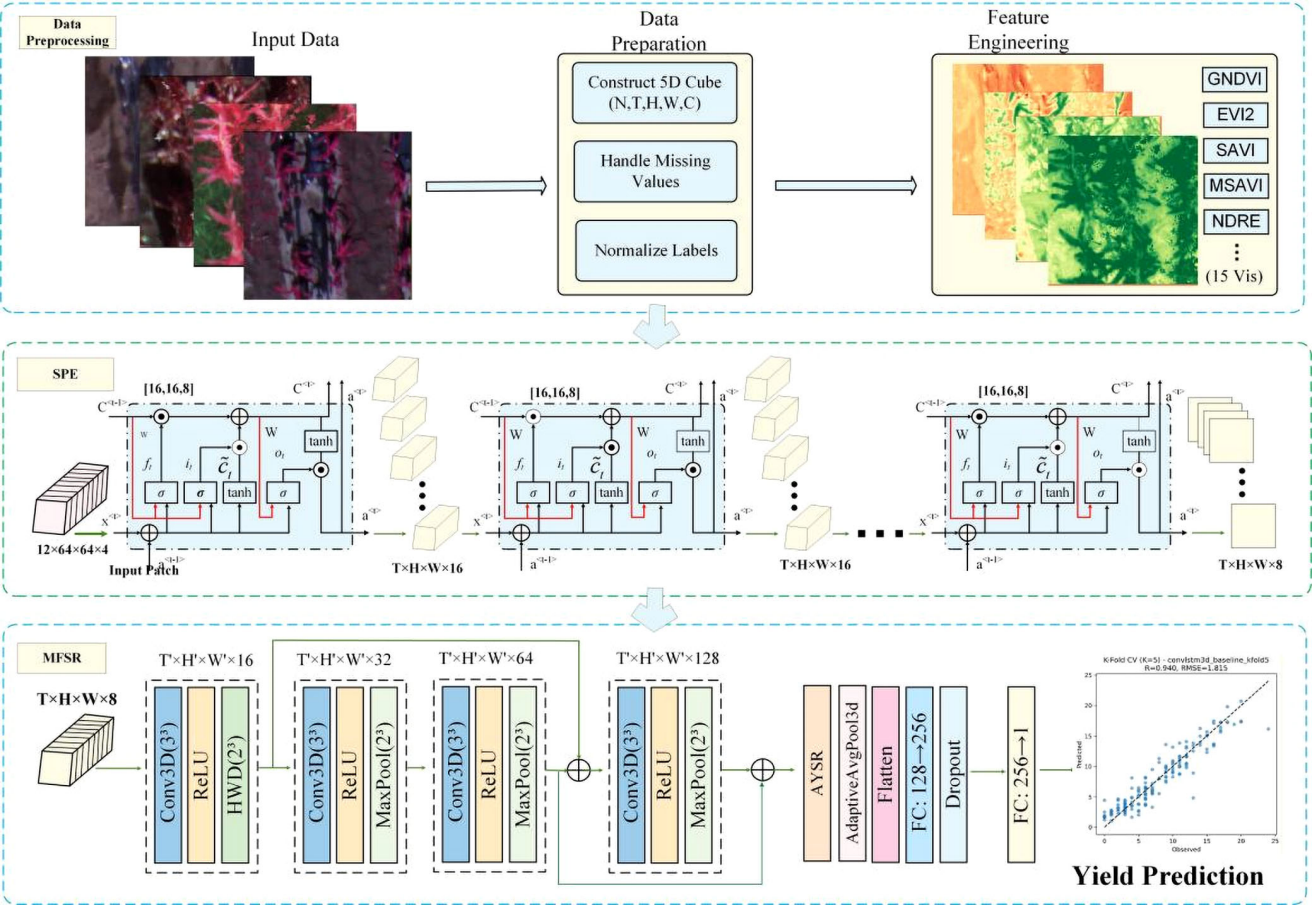
Overall architecture of the proposed WaveST-Yield model, integrating the Spatio-Temporal Phenology Encoder (SPE), 3D-CNN with Multiscale Frequency-Spatial Refiner (MFSR), and Adaptive Yield-Sensitive Re-calibrator (AYSR).

To address the shortcomings of existing UAV-based multispectral maize yield prediction methods, including insufficient mining of multispectral temporal features, ineffective separation of image details from noise, and inadequate response to yield-sensitive features that limit prediction accuracy for practical use, this study designs the WaveST-Yield hybrid model, whose structure is shown in **Figure 3**.

First, UAV multispectral data with high spatio-temporal-spectral consistency is acquired through systematic collection and preprocessing. The ConvLSTM-based Spatio-Temporal Phenology Encoder (SPE) is adopted first to capture temporal dynamic patterns and spatio-temporal correlations of multispectral data during the entire maize growth cycle, enhancing data temporal characteristics for subsequent deep feature learning. Subsequently, the data with enhanced temporal characteristics is input into the improved 3D-CNN for feature extraction. The first-layer downsampling of traditional 3D-CNN is replaced by HWD-based Multiscale Frequency-Spatial Refiner (MFSR) to preserve image details, decouple noise and prevent early loss of key maize physiological features. After four layers of feature extraction, CBAM-based Adaptive Yield-Sensitive Re-calibrator (AYSR) is embedded post feature extraction to enhance yield-sensitive feature extraction and suppress irrelevant background interference.

#### 2.2.1 SPE

To capture the temporal growth dynamics of maize throughout the whole growth period, the SPE based on ConvLSTM (Hochreiter and Schmidhuber, 1997) is employed as the temporal sequence encoder at the front end of the model, whose structure is shown in **Figure 3**. ConvLSTM replaces the linear operations of traditional LSTM with convolution operations (Zhang et al., 2026), which enables SPE to preserve the spatial structure of the input multispectral data while effectively modeling the temporal dependencies of maize growth. Specifically, the update process of ConvLSTM is expressed in Equation 1:

(1) $$H_{\text{t}},C_{t}=ConvLSTM\left(X_{t},H_{t-1},C_{t-1}\right)$$

where $H_{\text{t}}$ and $C_{\text{t}}$ are the hidden state and cell state at time step t, respectively, and $X_{\text{t}}$ is the input data at time t. Through the multi-layer stacked ConvLSTM network, the model can abstract temporal information layer by layer, and finally obtain temporal features with a dimension of T×H×W×8. Here, T represents the time step, H and W are the spatial dimensions, and 8 is the number of initial feature channels.

SPE first completes lightweight encoding and dimensionality reduction in the temporal dimension, which significantly reduces the computational cost of the subsequent 3D-CNN (Ji et al., 2013). Meanwhile, the lightweight 2D convolutional gating structure replaces the high-cost process of implicit temporal learning by 3D-CNN, further optimizing the model’s computational efficiency.

#### 2.2.2 MFSR

In CNN-based feature dimensionality reduction, traditional max pooling and average pooling often cause loss of discriminative details and fine-grained local structures, because they can lead to the loss of crucial spatial details and textural characteristics (El-Khamy et al., 2025; Zhao and Zhang, 2024) and are typically fixed rather than learnable (Shadoul et al., 2025). Haar Wavelet Downsampling (HWD) (Xu et al., 2023) provides a theoretically lossless alternative solution, which embeds Discrete Wavelet Transform (DWT) into the forward propagation of the network. It performs orthogonal frequency-domain decomposition on the input 2D feature maps, resulting in a low-frequency approximate subband (LL) that reflects the global smooth structure, and three high-frequency detail subbands (HL, LH, HH) that capture edge and detail information in horizontal, vertical, and diagonal directions respectively. This mechanism can retain the original feature information completely in the extended channel dimension while halving the spatial resolution, thereby realizing frequency-domain decoupling and lossless compression of features and providing support for subsequent high-quality feature extraction.

Introducing this frequency-domain decoupling downsampling mechanism into the maize multispectral spatiotemporal feature extraction framework has clear agricultural phenomics significance. In maize multispectral UAV images, the overall canopy morphology, ridge distribution, and soil background form a stable low-frequency structure, while leaf edges, local growth heterogeneity, and the high-frequency response of specific bands to chlorophyll are key fine-grained phenotypic clues determining maize yield. Traditional spatial pooling blurs the frequency-domain boundary, leading to aliasing between background noise and crop signals; Among various wavelet bases, the Haar wavelet is widely used for efficiently decomposing features into approximate and detail components (Zhang et al., 2024). Haar wavelet transform can explicitly separate features of different frequencies, ensuring that the model accurately anchors the canopy baseline and captures local spectral-spatial variations at the initial stage of feature compression, which is crucial for yield prediction relying on micro-phenotypic differences.

**Figure 4:**
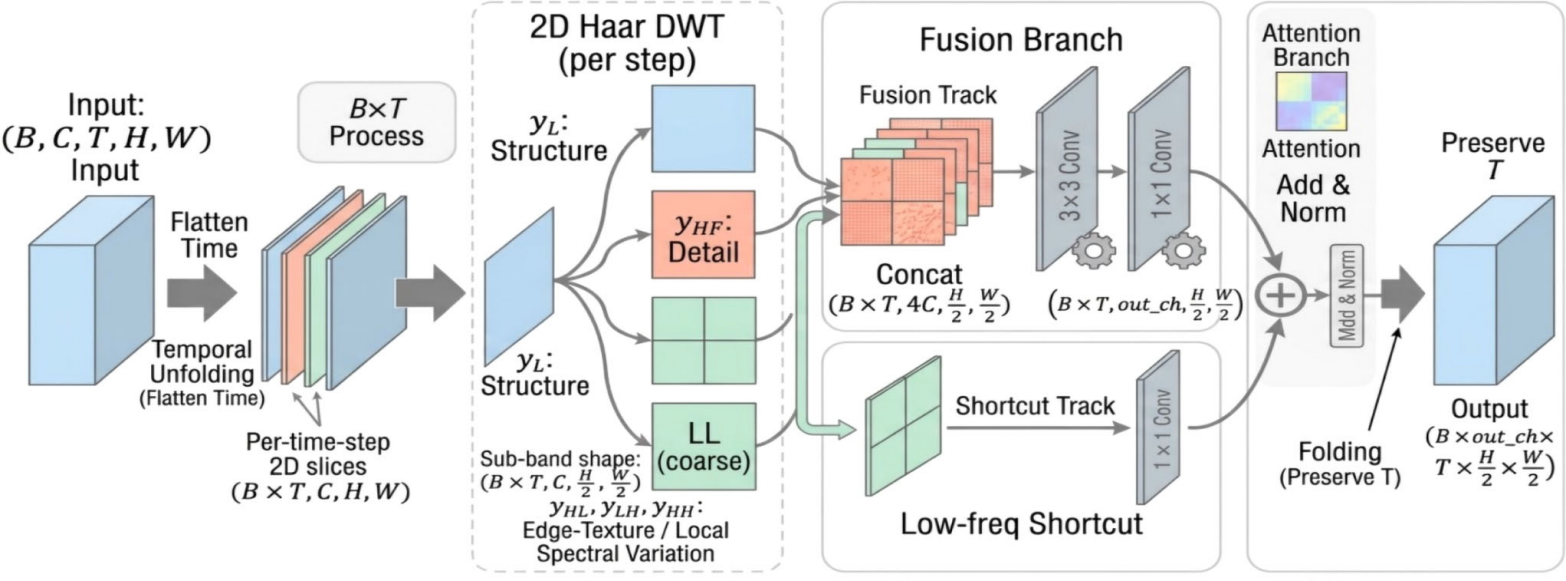
Detailed network architecture of the multiscale frequency-spatial refiner (MFSR) module based on 2D Haar discrete wavelet transform (DWT) and dual-stream fusion branch.

To adapt to the 3D spatiotemporal data stream in this study, after the temporal sequence encoding (SPE) module at the front end of the model, a multi-scale frequency-spatial refiner (MFSR) is designed based on HWD transform, whose structure is shown in **Figure 4**. The MFSR first unfolds the spatiotemporal tensor into 2D slices along the time axis and performs 2D Haar decomposition frame by frame to avoid interference from the time dimension; then it constructs a dual-stream architecture, where the main fusion branch completes cross-frequency compression and texture extraction of high and low frequency subbands through a convolution sequence, and the low-frequency shortcut branch maps the LL subband to provide a structural benchmark; finally, it restores the 5D tensor through dynamic aggregation and time folding. This design preserves the original phenological time length while completing frequency-domain purification and spatial refinement of phenotypic information in advance, laying a high-quality foundation for subsequent feature extraction.

#### 2.2.3 AYSR

The Convolutional Block Attention Module (CBAM) is a lightweight and universal feed-forward convolutional neural network attention mechanism. Inspired by the attention allocation law of the human visual system, it dynamically recalibrates feature maps by decoupling the attention weight inference processes of the channel and spatial dimensions (Woo et al., 2018). The channel attention branch aggregates spatial information through global average pooling and max pooling, and generates channel weights via a multi-layer perceptron (MLP) to distinguish the importance of each feature channel; the spatial attention branch generates a 2D weight map through convolutional layers based on pooling operations along the channel dimension, thereby locating key spatial regions in the image. The serial combination of these two branches effectively suppresses background noise and guides the network to focus on the most discriminative target feature regions.

After front-end SPE encoding and mid-end 3D-CNN spatiotemporal feature extraction, the feature cube generated by the model is highly abstract, integrating deeply coupled features of temporal phenological evolution, multispectral responses, and spatial topology information. During the entire growth period of maize, the contributions of different time steps, multispectral channels, and spatial plots to the final yield vary significantly. Undifferentiated global pooling or fully connected regression will dilute key features with redundant spatiotemporal background information, while the CBAM attention mechanism can achieve dynamic identification and weighting in feature depth, spatial position, and time span, thereby filtering redundant phenological noise and enhancing the model’s focus on core yield phenotypes.

**Figure 5:**
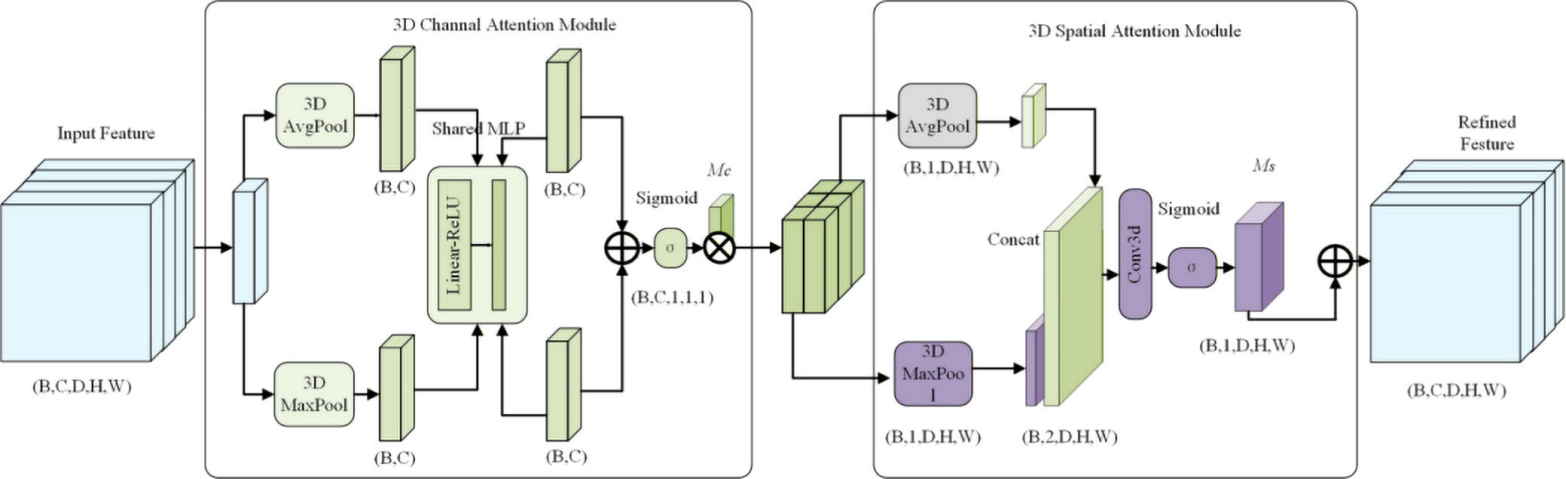
Detailed network structure of the adaptive yield-sensitive re-calibrator (AYSR) module, featuring 3D channel attention and 3D spatial attention modules for yield-sensitive feature enhancement.

To address the limitation that traditional 2D CBAM cannot process time-dimensional information, this study extends it to three dimensions and designs an AYSR module adapted to 5D tensors, whose structure is shown in **Figure 5**. In the channel attention part, the original 2D spatial pooling is upgraded to 3D spatiotemporal global pooling, enabling the module to comprehensively evaluate the contribution of each abstract feature channel to yield across the entire growth period and spatial scope. In the spatial attention part, it is extended to spatiotemporal attention; after pooling along the channel dimension, a 3D convolution kernel is used to generate a 3D attention heatmap, which can not only locate key spatial regions but also identify critical phenological stages that have a significant impact on yield. Through continuous multiplicative recalibration in both channel and spatiotemporal dimensions, the AYSR module greatly enhances the discriminability and robustness of the final yield regression features.

### 2.3 Model training and parameter setting

To improve the generalization ability of the model during the training phase, regularization methods including Dropout and L2 Regularization (Clara et al., 2024) were enabled, and the Adam optimizer was adopted to optimize the model parameters. The learning rate was tuned based on the validation set, and an early stopping strategy was adjusted to avoid overfitting. In terms of hyperparameter selection, this study adopted a combination of grid search and random search to select the optimal hyperparameter combination, with R² and RMSE of the validation set as the key evaluation metrics.

All experiments were conducted under the Windows operating system. The hardware platform consisted of an NVIDIA graphics card and 32 GB RAM. The deep learning model was implemented using the PyTorch framework, while traditional machine learning models were implemented using the scikit-learn and XGBoost libraries. To ensure the reproducibility of the experimental results, a fixed random seed of 42 was used for all training processes. Detailed parameters are shown in **Table 2**.

**Table 2:** Experimental environment configuration.

Configuration name	Version and model
GPU Model & Quantity	NVIDIA GeForce RTX 4090D
CPU Model	Intel (R) Xeon (R) Platinum 8270CPU@2. 70GHz
System RAM	128.0GB
Operating System	Windows 11 Home Chinese Edition(22H2build)
Deep Learning Framework	torch2.6.0+cu124
CUDA with cuDNN version	CUDA12.4+cuDNN8.9.7

To avoid data leakage and ensure the reliability of experimental results, 5-fold cross-validation was adopted as the primary evaluation strategy (Zewdie et al., 2025). In each fold, 129 samples were used for training and 32 for validation. During model training, data augmentation was applied to the training set, and the model was evaluated on an independent validation set. Specifically, Field 1 was used for model training and cross-validation, while Field 2 served as the external validation set to assess the model’s generalization ability. To further verify this ability, cross-filed external validation was conducted between the two independent experimental plots: the model was trained on one field and tested on the other, with reverse validation also implemented. This scheme enables the comparison of model robustness and generalization ability under different environmental conditions, and verifies its adaptability to unseen fields.

### 2.4 Evaluation metrics

Based on 5-fold cross-validation, model evaluation was performed on the validation set, and metrics including the Coefficient of Determination (R²), Root Mean Square Error (RMSE), Mean Absolute Error (MAE), Mean Absolute Percentage Error (MAPE), and Pearson Correlation Coefficient (PCC) were calculated to characterize the prediction accuracy and stability of the model. The model performance of each fold was obtained through cross-validation, and the results of all folds were statistically analyzed and presented as mean ± standard deviation. The formulas of the specific evaluation metrics are presented in Equations 2–6 as follows:

(2) $${\text{R}}^{2}=1-\frac{\sum_{\text{i}=1}^{\text{N}}{\left(y_{i}-{\overset{\wedge }{\text{y}}}_{i}\right)}^{2}}{\sum_{i=1}^{N}{\left(y_{i}-\bar{y}\right)}^{2}}$$

where ${\text{y}}_{i}$ is the observed value of the i-th sample, ${\hat{\text{y}}}_{i}$ is the predicted value of the i-th sample, $\bar{\text{y}}$is the mean of the observed values of all samples, and N is the total number of samples.

(3) $$\text{RMSE}=\sqrt{\frac{1}{\text{N}}\sum_{i=1}^{N}({\text{y}}_{i}-{\hat{\text{y}}}_{i}{)}^{2}}$$

where ${\text{y}}_{i}$ and ${\hat{\text{y}}}_{i}$ are the observed value and predicted value of the i-th sample, respectively, and N is the total number of samples.

(4) $$\text{MAE}=\frac{1}{\text{N}}\sum_{i=1}^{\text{N}}|y_{i}-{\hat{\text{y}}}_{i}|$$

where ${\text{y}}_{i}$ and ${\hat{\text{y}}}_{i}$ are the observed value and predicted value of the i-th sample, respectively, and N is the total number of samples.

(5) $$\text{MAPE}=\frac{1}{\text{N}}\sum_{i=1}^{N}\left|\frac{y_{i}-{\hat{\text{y}}}_{i}}{y_{i}}\right|\times 100\%$$

where ${\text{y}}_{i}$ and ${\hat{\text{y}}}_{i}$ are the observed value and predicted value of the i-th sample, respectively, and N is the total number of samples.

(6) $$\text{PCC}=\frac{\sum_{i=1}^{N}\left({\text{y}}_{i}{-}_{\bar{\text{y}}})({\hat{y}}_{i}-\bar{\hat{y}}\right)}{\sqrt{\sum_{i=1}^{N}({\text{y}}_{i}{-}_{\bar{\text{y}}}{)}^{2}\sum_{i=1}^{N}{\left({\hat{y}}_{i}-\bar{\hat{y}}\right)}^{2}}}$$

where $\bar{\text{y}}$ and $\hat{\bar{\text{y}}}$ are the mean of the observed values and the mean of the predicted values, respectively, ${\text{y}}_{i}$ and ${\hat{\text{y}}}_{i}$ are the observed value and predicted value of the i-th sample, respectively, and N is the total number of samples.

## 3 Experimental results and analysis

### 3.1 Ablation study

#### 3.1.1 Field 1

To verify the synergistic effect of each core module in the WaveST-Yield model, 6 groups of ablation experiments were designed based on the data of Field 1 to evaluate the impact of a single module and module combinations on the model’s prediction performance, with the experimental results shown in **Table 3**. Herein, A represents the SPE, B represents the original 3D-CNN, C represents the MFSR, and D represents the AYSR.

**Table 3:** Ablation study table of Field 1.

Group	Module				Mean ± std					Overall				
	A	B	C	D	R²	RMSE	MAE	MAPE%	PCC	R²	RMSE	MAE	MAPE%	PCC
1	✓				0.833 ± 0.088	1.983 ± 0.537	1.5268 ± 0.400	23.407 ± 5.849	0.922 ± 0.038	0.85	2.053	1.527	23.599	0.926
2		✓			0.649 ± 0.109	2.939 ± 0.219	2.333 ± 0.212	40.752 ± 8.47	0.846 ± 0.055	0.689	2.96	2.336	40.861	0.856
3	✓	✓			0.855 ± 0.057	1.887 ± 0.276	1.429 ± 0.113	26.966 ± 8.737	0.925 ± 0.032	0.87	1.911	1.43	26.911	0.933
4	✓	✓	✓		0.855 ± 0.078	1.878 ± 0.373	1.386 ± 0.157	24.756 ± 8.839	0.925 ± 0.043	0.871	1.91	1.388	24.71	0.934
5	✓	✓		✓	0.759 ± 0.106	2.398 ± 0.432	1.874 ± 0.446	37.499 ± 17.987	0.886 ± 0.047	0.789	2.436	1.873	38.009	0.894
6	✓	✓	✓	✓	0.870 ± 0.058	1.767 ± 0.408	1.347 ± 0.270	25.162 ± 9.260	0.934 ± 0.031	0.883	1.814	1.347	25.05	0.941

It can be seen that the prediction performance of the experimental group with only the SPE module (Group 1) is significantly better than that of the experimental group with only the original 3D-CNN module (Group 2); the prediction performance of the experimental group with the combination of SPE module and original 3D-CNN module (Group 3) is further improved compared with the single-module group; after adding the MFSR module (Group 4), the model performance remains stable and the error index is slightly optimized; when the AYSR module is embedded without the MFSR module (Group 5), the prediction performance is significantly reduced; the complete model with all modules working together (Group 6) achieves the optimal prediction effect. Therefore, the SPE module is the core foundation for improving the prediction accuracy, the MFSR module can effectively realize noise decoupling and detail preservation without reducing the model performance, the AYSR module needs to play a role under the coordination of the MFSR module, and the synergistic effect of all modules can maximize the prediction performance of the model.

#### 3.1.2 Field 2

The above six groups of ablation experiments were also verified on Field 2, and the experimental results are shown in **Table 4**. Although the overall prediction indicators are slightly lower than those of Field 1, the interaction law between different modules is consistent with that of Field 1, which effectively verifies the stability and reliability of the synergistic effect of each core module.

**Table 4:** Ablation study table of Field 2.

Group	Module				Mean ± std						Overall						
	A	B	C	D		R²	RMSE	MAE	MAPE%	PCC		R²	RMSE	MAE	MAPE%	PCC	
1	✓					0.688 ± 0.074	2.012 ± 0.286	1.602 ± 0.221	33.800 ± 5.100	0.844 ± 0.040		0.691	2.031	1.608	33.72	0.847	
2		✓				0.682 ± 0.078	2.024 ± 0.293	1.615 ± 0.216	33.458 ± 4.596	0.840 ± 0.042		0.685	2.045	1.615	33.388	0.829	
3	✓	✓				0.728 ± 0.067	1.936 ± 0.257	1.522 ± 0.186	31.482 ± 7.903	0.856 ± 0.041		0.734	1.914	1.509	31.08	0.862	
4	✓	✓	✓			0.761 ± 0.072	1.744 ± 0.261	1.370 ± 0.200	33.437 ± 18.192	0.876 ± 0.042		0.766	1.763	1.37	33.33	0.876	
5	✓	✓		✓		0.709 ± 0.071	2.031 ± 0.294	1.595 ± 0.214	34.276 ± 8.115	0.848 ± 0.044		0.714	2.006	1.587	34.02	0.853	
6	✓	✓	✓	✓		0.773 ± 0.080	1.694 ± 0.342	1.350 ± 0.262	29.471 ± 11.501	0.883 ± 0.046		0.775	1.729	1.35	29.36	0.881	

### 3.2 Multi-model comparison experiment analysis

#### 3.2.1 Field 1

The WaveST-Yield model is compared with traditional machine learning models as well as typical deep learning models including ConvLSTM, 3D-CNN and iTransformer, and the experimental results based on the data of Field 1 are shown in **Table 5**. It can be observed that the improved WaveST-Yield model demonstrates superior comprehensive performance. In terms of prediction accuracy, its Overall R² is significantly higher than all the compared models; in terms of error control, its RMSE, MAE and MAPE are all better than others, and the MAPE is about 6 percentage points lower than that of the optimal traditional model. In terms of stability and reliability, its PCC (0.941) is the highest among all models, showing better correlation and stability. Through temporal coding, detail preservation and other optimization measures, the model achieves more accurate and stable yield prediction results, which can better meet the practical needs of plot-scale yield monitoring and effectively avoid the limitations of traditional machine learning models in handling complex and heterogeneous scenarios.

**Table 5:** Multi-model comparison experimental results of Field 1.

Model	Mean ± std					Overall				
	R²	RMSE	MAE	MAPE%	PCC	R²	RMSE	MAE	MAPE%	PCC
XGB	0.775 ± 0.042	2.384 ± 0.184	1.874 ± 0.149	38.441 ± 16.880	0.893 ± 0.036	0.797	2.392	1.875	38.396	0.901
RF	0.726 ± 0.045	2.633 ± 0.158	2.019 ± 0.161	39.780 ± 17.810	0.866 ± 0.038	0.753	2.638	2.019	39.783	0.872
Ridge	0.824 ± 0.068	2.062 ± 0.291	1.583 ± 0.249	30.958 ± 10.269	0.914 ± 0.031	0.846	2.082	1.582	31.090	0.921
LR	0.774 ± 0.028	2.403 ± 0.181	1.874 ± 0.187	39.669 ± 16.357	0.906 ± 0.019	0.794	2.411	1.874	39.643	0.913
ConvLSTM	0.833 ± 0.088	1.983 ± 0.537	1.5268 ± 0.400	23.407 ± 5.849	0.922 ± 0.038	0.85	2.053	1.527	23.599	0.926
3D-CNN	0.649 ± 0.109	2.939 ± 0.219	2.333 ± 0.212	40.752 ± 8.47	0.846 ± 0.055	0.689	2.96	2.336	40.861	0.856
iTransformer	0.638 ± 0.186	2.926 ± 0.914	2.379 ± 0.786	42.783 ± 11.62	0.836 ± 0.09	0.672	3.040	2.382	42.794	0.839
WaveST-Yield	0.870 ± 0.058	1.767 ± 0.408	1.347 ± 0.270	25.162 ± 9.260	0.934 ± 0.031	0.883	1.814	1.347	25.050	0.941

#### 3.2.2 Field 2

Multi-model comparison experiments were simultaneously conducted on Field 2, and the experimental results are shown in **Table 6**. Consistent with the experimental results of Field 1, the WaveST-Yield model achieves the optimal comprehensive performance, which is superior to other models in terms of R², error control and stability. This effectively verifies the universality and reliability of the model across different plots and scenarios, providing stable and reliable technical support for plot-scale maize yield prediction.

**Table 6:** Multi-model comparison experimental results of Field 2.

Model	Mean ± std					Overall				
	R²	RMSE	MAE	MAPE%	PCC	R²	RMSE	MAE	MAPE%	PCC
XGB	0.604 ± 0.047	2.272 ± 0.163	1.784 ± 0.116	40.169 ± 12.213	0.796 ± 0.037	0.609	2.277	1.784	40.113	0.792
RF	0.538 ± 0.066	2.450 ± 0.208	1.937 ± 0.172	43.831 ± 13.462	0.767 ± 0.057	0.545	2.459	1.937	43.74	0.755
Ridge	0.653 ± 0.130	2.098 ± 0.390	1.535 ± 0.268	39.872 ± 22.221	0.820 ± 0.088	0.657	2.134	1.535	39.68	0.817
LR	0.496 ± 0.063	2.565 ± 0.236	2.042 ± 0.165	49.454 ± 15.537	0.767 ± 0.051	0.501	2.575	2.042	49.38	0.758
ConvLSTM	0.688 ± 0.074	2.012 ± 0.286	1.602 ± 0.221	33.800 ± 5.100	0.844 ± 0.040	0.691	2.031	1.608	33.72	0.847
3D-CNN	0.682 ± 0.078	2.024 ± 0.293	1.615 ± 0.216	33.458 ± 4.596	0.840 ± 0.042	0.685	2.045	1.615	33.388	0.829
iTransformer	0.632 ± 0.025	2.189 ± 0.103	1.664 ± 0.092	34.148% ± 11.403	0.807 ± 0.017	0.639	2.191	1.664	34.088	0.804
WaveST-Yield	0.773 ± 0.080	1.694 ± 0.342	1.350 ± 0.262	29.471 ± 11.501	0.883 ± 0.046	0.775	1.729	1.35	29.36	0.881

### 3.3 Statistical stability analysis

To further verify the statistical stability and reliability of the WaveST-Yield model across different plots, supplementary statistical analysis was performed on its prediction results in Field 1 and Field 2. Based on a 5-fold cross-validation design, each fold was repeated three times to minimize random experimental errors. Key evaluation metrics were summarized from the total experimental data, and 95% confidence interval (CI) were calculated to quantitatively reflect the model’s performance fluctuation and reliability, with results shown in **Table 7**.

**Table 7:** Statistical stability analysis of the WaveST-Yield model in Field 1 and Field 2 based on repeated five-fold cross-validation.

Metric	Field 1				Field 2			
	Mean	Std	95% CI lower	95% CI upper	Mean	Std	95% CI lower	95% CI upper
R^2^	0.888	0.051	0.860	0.917	0.764	0.0850	0.727	0.801
RMSE	1.670	0.368	1.467	1.874	1.748	0.309	1.577	1.919
MAE	1.287	0.250	1.148	1.425	1.450	0.239	1.318	1.582
MAPE%	22.495	6.867	18.692	26.298	30.744	10.881	24.719	36.770
PCC	0.945	0.026	0.930	0.959	0.869	0.051	0.841	0.897

**Table 7** shows that the WaveST-Yield model maintains good statistical stability in both fields. In Field 1, it exhibits excellent stability: the R² mean is 0.888, error metrics fluctuate slightly, and the PCC mean reaches 0.945 with a narrow confidence interval, indicating high consistency and correlation. In Field 2, although the overall performance is slightly lower and error metrics fluctuate more, this aligns with the earlier conclusion that Field 2 has higher heterogeneity. Notably, all metrics have narrow 95% CIs, confirming the model’s resistance to random factors and reproducibility.

### 3.4 Visualized analysis

#### 3.4.1 Analysis of predicted vs. observed values

**Figure 6:**
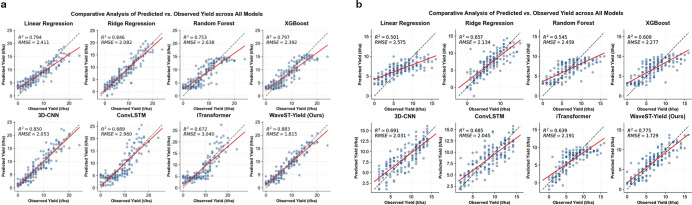
Comparative analysis of predicted versus observed maize yield for different models using 5-fold cross-validation. **(a)** Field 1 results; **(b)** Field 2 results.

To quantitatively verify the prediction consistency of each model, 5-fold cross-validation was employed to plot scatter diagrams of predicted vs. observed values for the two study fields, with the 1:1 reference line representing the ideal prediction state. Results for Field 1 are shown in **Figure 6a**, and results for Field 2 are presented in **Figure 6b**.

The figure shows that, WaveST-Yield shows a high degree of alignment with the 1:1 reference line in both Field 1 and Field 2, with data points tightly clustered and minimal systematic bias. ConvLSTM and 3D-CNN exhibit similar performance but show slight dispersion in the high observed value range. This indicates single temporal or spatiotemporal modeling fails to characterize complex nonlinear relationships in high-yield samples. Notably, iTransformer performs the worst among all models, with the most scattered points and the largest systematic deviation from the reference line. By contrast, WaveST-Yield avoids such dispersion via SPE full-growth-period temporal encoding, MFSR frequency-domain detail preservation and AYSR yield-sensitive feature recalibration, enhancing its sensitivity to subtle high-yield phenotypic differences.

Traditional machine learning models (XGB, RF) and linear models (LR, Ridge) display more pronounced deviations: XGB and RF have greater data dispersion, while LR and Ridge exhibit obvious under or overestimation in certain ranges, especially in Field 2. Wider scattering in Field 2 stems from higher soil, microclimate and phenological heterogeneity. However, WaveST-Yield with its 3D spatiotemporal feature extraction capability, effectively captures complex nonlinear relationships in the data and outperforms shallow models and other deep learning architectures in terms of prediction accuracy and stability.

#### 3.4.2 Model performance hierarchy analysis

**Figure 7:**
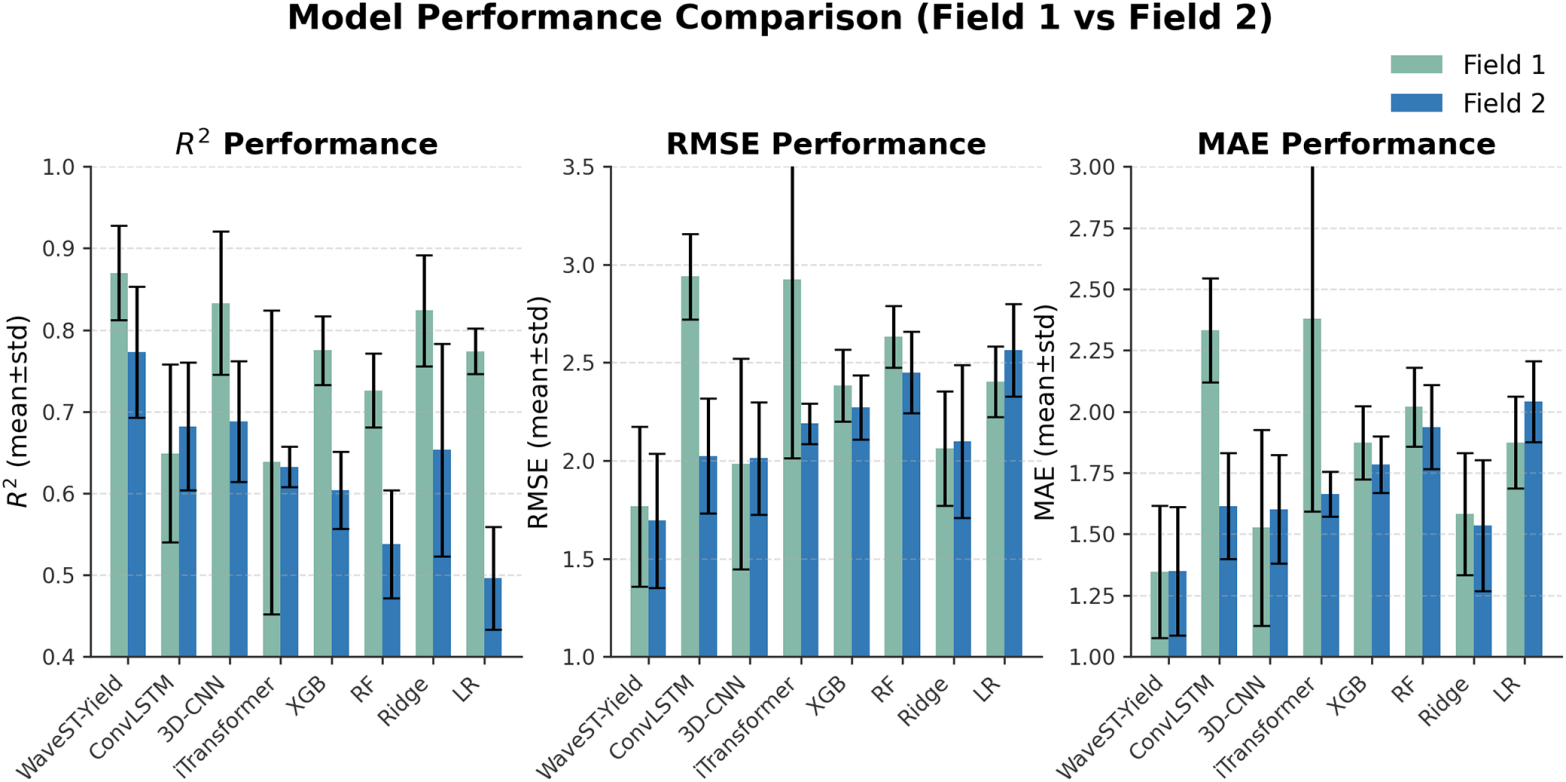
Cross-validation performance comparison (R^2^, RMSE, and MAE) across different models for both experimental fields. Error bars represent the standard deviation of the 5-fold cross-validation results.

To more intuitively compare the prediction accuracy and stability of different models in the two experimental fields, this study plotted model performance comparison charts, as shown in **Figure 7**. The mean and standard deviation of the five-fold cross-validation results were simultaneously displayed using error bars.

The figure shows that WaveST-Yield achieved the best performance across both fields, with higher overall R2 and lower RMSE and MAE values. Its smaller error bars represent lower cross-validation fluctuation and stronger anti-interference robustness. 3D-CNN and Ridge performed well in Field 1 but showed varying degrees of decline in Field 2, reflecting their relatively limited cross-field generalization ability. This performance degradation is mainly attributed to their poor adaptability to elevated spatiotemporal phenotypic heterogeneity between fields. By comparison, iTransformer, XGB, RF, and LR all exhibited weaker performance, with larger fluctuations, higher errors, and lower goodness of fit, especially in Field 2, indicating they fail to adequately capture complex spatiotemporal phenotypic information.

#### 3.4.3 Multi-dimensional comprehensive performance analysis of models

**Figure 8:**
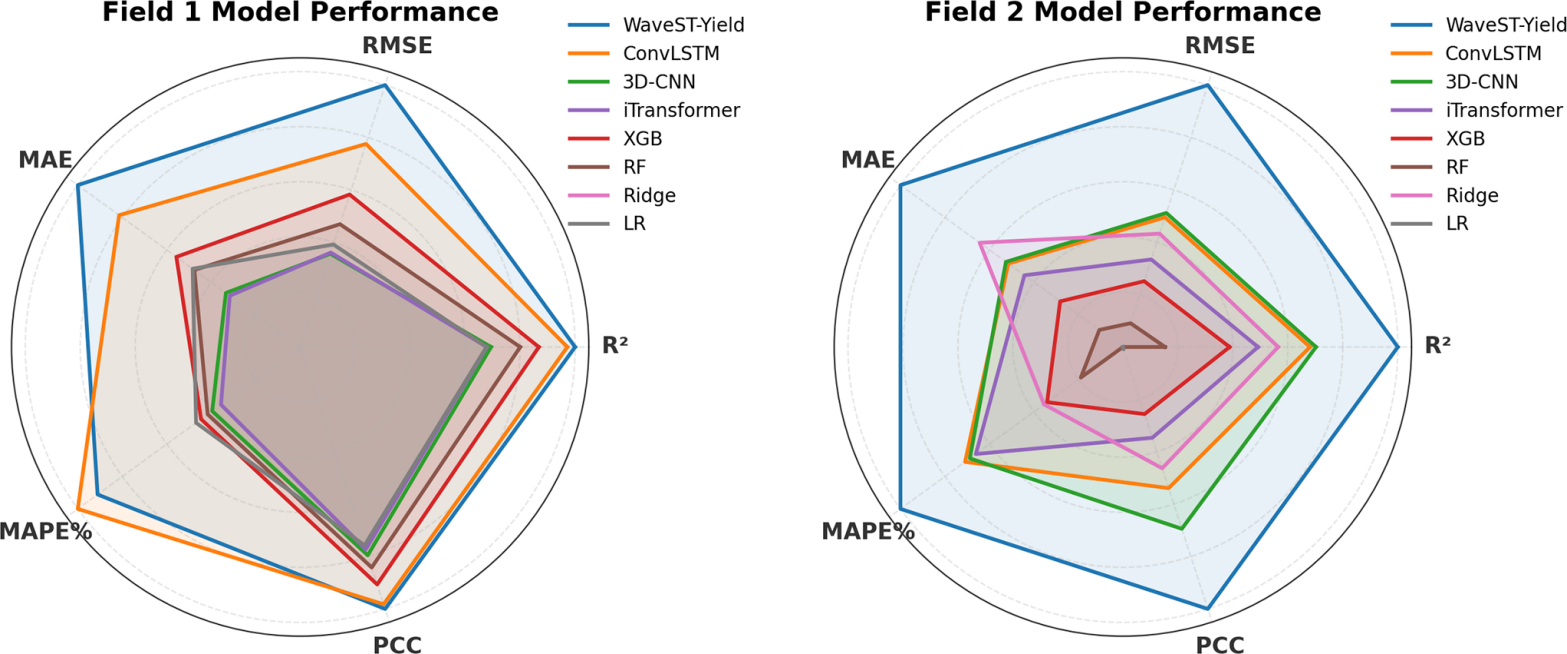
Multi-dimensional comprehensive performance evaluation of the prediction models using radar charts across five evaluation metrics (R^2^, RMSE, MAE, MAPE%, and PCC).

Since the optimization directions of various evaluation metrics are inconsistent, to achieve a unified quantitative comparison, this study normalized the original data and took the reciprocal of the smaller-the-better metrics to unify the optimization directions. Finally, the comprehensive performance of each model across different metric dimensions was visually presented through radar charts, as shown in **Figure 8**.

In Field 1, WaveST-Yield outperforms other models significantly in terms of MAE and RMSE; although its MAPE is slightly lower than that of ConvLSTM, its overall performance still exhibits clear advantages. This multi-metric dominance reflects a balanced trade-off among fitting ability, error control and cross-field stability. Especially in the experimental results of Field 2, all other models show significant decreases in various indicators, while WaveST-Yield maintains its dominant advantage. This confirms its core modules effectively suppress heterogeneity-induced performance degradation.

### 3.5 Experimental analysis of hyperparameter tuning

**Figure 9:**
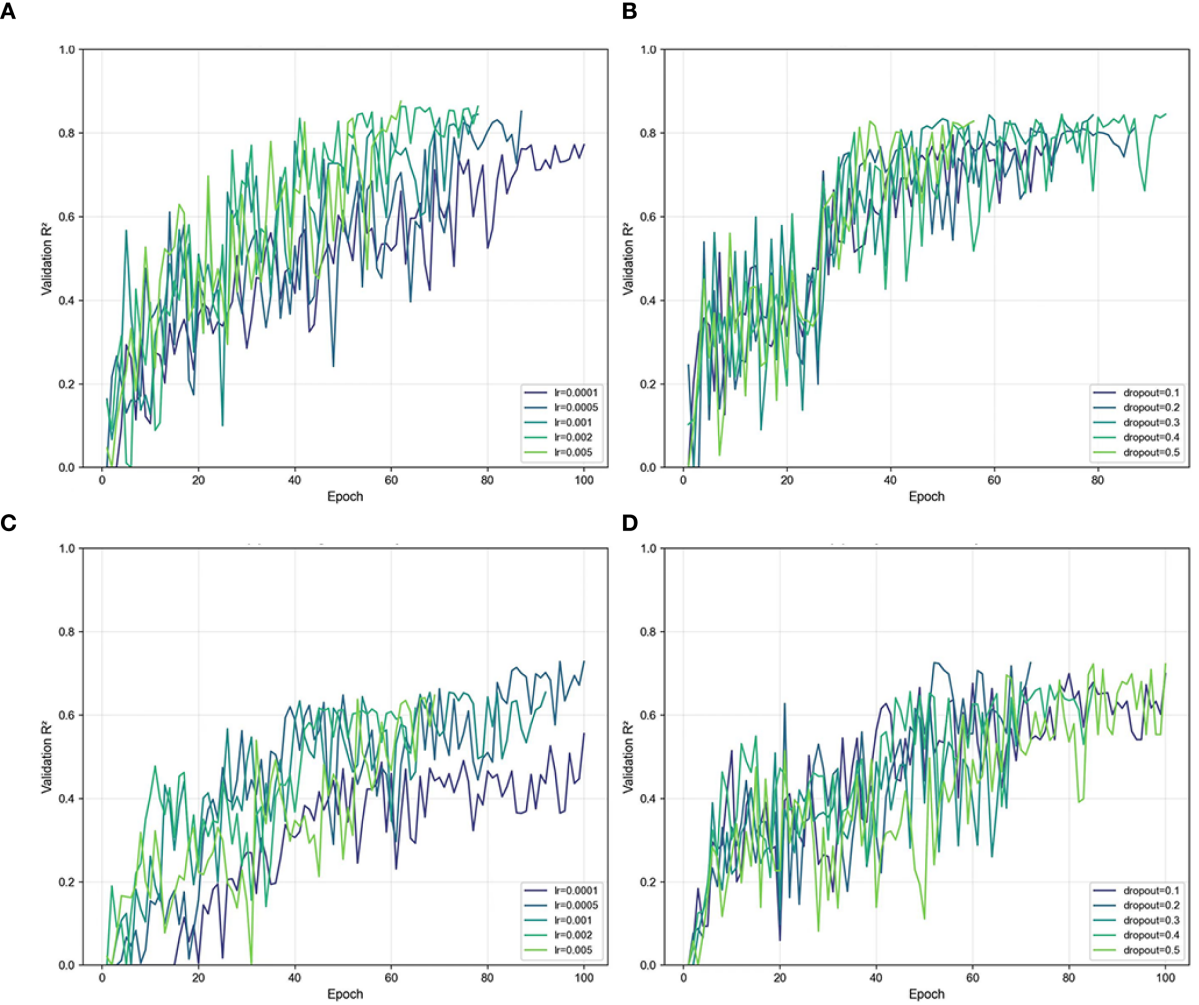
Hyperparameter tuning experimental results of the WaveST-Yield model for Field 1 **(A)** and Field 2 **(B)**. Each panel includes: **(A)** learning rate comparison, **(B)** dropout rate comparison, **(C)** batch size performance, and **(D)** model architecture performance analysis.

**Figure 10:**
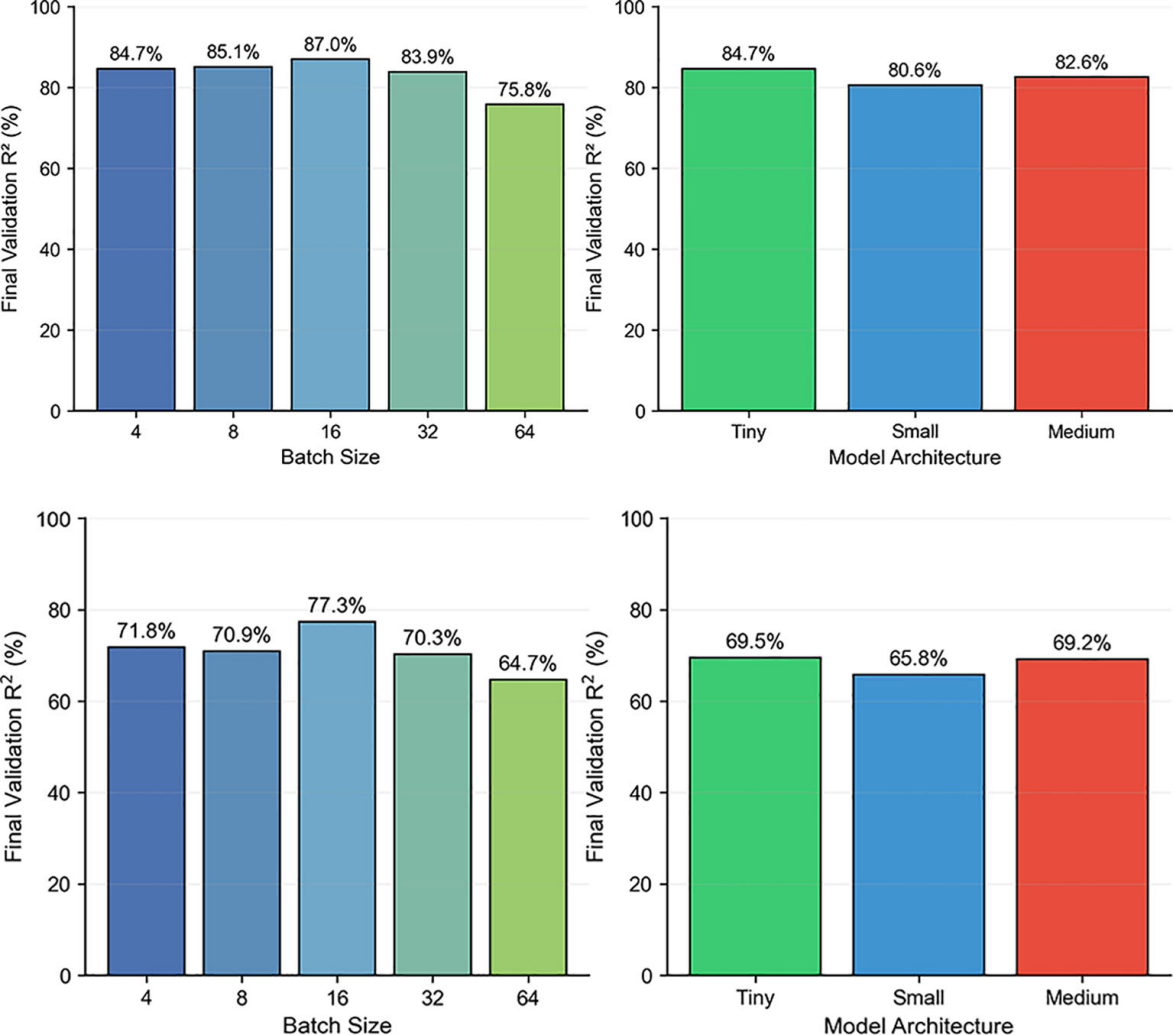
Hyperparameter sensitivity analysis of the WaveST-Yield model under different batch sizes and model architectures in Field 1 and Field 2.

Hyperparameters are critical to the performance of deep learning models, and their values determine the convergence speed, fitting ability, and generalization of the model. Improper settings can lead to overfitting, slow convergence, or gradient oscillation, limiting the prediction performance of the WaveST-Yield model. To determine the optimal hyperparameter combination and improve the prediction accuracy and stability of the model, this study systematically evaluates the key hyperparameters of the model, and the experimental results are shown in **Figure 9**, **10**. **Figure 9** shows the effects of learning rate and dropout rate, while **Figure 10** shows the hyperparameter sensitivity analysis under different batch sizes and model architectures in Field 1 and Field 2.

For Field 1, a learning rate of 0.001 ensures steady convergence, a dropout rate of 0.2–0.3 balances model fitting and generalization, a batch size of 16 yields the highest validation R2 (87.0%), and the Tiny architecture achieves the best performance (84.7%) due to its lightweight feature extraction capability. For Field 2, the overall performance declines due to higher data heterogeneity. The optimal learning rate (0.001) and dropout range (0.2–0.3) remain consistent, but the peak validation R2 (77.3%) at batch size 16 and the Tiny architecture’s performance (69.5%) are significantly lower than those in Field 1, indicating the need for further hyperparameter optimization for the new dataset. Consistent optimal hyperparameters verify the model’s adaptability to multi-temporal/multi-channel inputs; performance reduction corresponds to Field 2’s stronger small-sample cross-field heterogeneity.

## 4 Discussion

Current research on maize yield estimation based on UAV remote sensing still faces several core limitations. Most studies take a small number of manually constructed vegetation indices such as NDVI and GNDVI as the core inputs. Even though Guo et al. optimized the feature system by fusing RGB and multispectral vegetation indices, these methods still heavily rely on manual feature engineering and fail to fully retain the spectral and spatial information of raw multispectral data. While Bao et al. (2024) and Kumar et al. (2023) verified the yield estimation value of multispectral data, they did not utilize the full-growth-period time-series data of maize, making it impossible to fully characterize the temporal growth dynamics of yield formation. Most studies such as those by Yan et al. (2026); Bao et al. (2025), and Wang et al. (2025) still rely on traditional machine learning algorithms, which makes it difficult to fully explore the deep nonlinear relationships between remote sensing data and yield, and the generalization ability of the models is significantly restricted by regional environments and growing conditions. Among existing deep learning-related studies, the models developed by Wang et al., (2023) lack mechanistic interpretability, the LSTM model used by Tian et al. (2021) has insufficient prediction accuracy and no generalization ability verification is conducted, and existing methods generally lack the organic integration of spectral-spatial-temporal three-dimensional features. As a result, the methodological system for yield estimation using maize multi-temporal multispectral data remains incomplete.

To address the limitations of existing UAV-based multispectral maize yield prediction models, including inadequate temporal feature mining, poor detail-noise separation, and weak sensitivity to yield-related features, this study proposes WaveST-Yield, a hybrid model with three core modules: SPE for capturing spatio-temporal features, HWD for image detail preservation, and AYSR for yield-sensitive feature enhancement. To ensure model reliability, five-fold cross-validation and cross-field validation are employed, with comparative experiments conducted against mainstream models.

The results of the ablation experiments confirm that the core modules of the WaveST-Yield model synergistically enhance spectral and spatial information extraction, improving model adaptability and yield prediction accuracy. Comparative experiments with multiple models further indicate that WaveST-Yield significantly outperforms traditional machine learning models and single-structure deep learning models in terms of prediction accuracy, stability, and generalization ability, reflecting the core value of spatiotemporal joint modeling in the analysis of the relationship between crop phenotypes and yield. The results of cross-plot external validation show that WaveST-Yield maintains stable performance in scenarios with significant differences in soil types and microclimate conditions, proving that the model has good environmental adaptability and cross-regional generalization ability. It can effectively break through the limitations of field heterogeneity and lay the foundation for the large-scale promotion of the model in actual agricultural production.

Compared with the original single 3D-CNN baseline, the proposed WaveST-Yield model improves the coefficient of determination by 19 points in Field 1 and 9 points in Field 2, with all error metrics significantly decreased. Compared with the single ConvLSTM baseline, the coefficient of determination is increased by 3 points in Field 1 and 4 points in Field 2. Meanwhile, WaveST-Yield surpasses all traditional machine learning methods including XGBoost, RF, Ridge and LR. Compared with the optimal traditional model, the coefficient of determination rises by 4 points in Field 1 and 12 points in Field 2, and the mean absolute percentage error is also greatly reduced. In general, the proposed model achieves greatly improved prediction accuracy, stability and generalization performance for maize yield prediction.

To further verify the robustness of the proposed model against variable imaging conditions, UAV imagery was collected under controlled conditions with manual adjustment to reduce shadows and optimize view geometry. Two geographically separated plots (16 km apart) with 161 subplots were established, and ground-truth yield values were verified via triple measurements using a milligram-precision balance. Data acquisition was conducted under sunny conditions from 10:00 to 14:00, which is typical and easily achievable in agricultural practice. Mutual cross-validation between the two plots was performed to avoid overfitting. Although model performance may degrade slightly under changed UAV platforms, illumination, shadows and view geometry, the adopted 3D-CNN and ConvLSTM framework together with normalization and spatial-spectral feature alignment exhibit strong robustness to imaging variations and effectively reduce cross-domain discrepancies.

Nevertheless, it should be noted that this study still has certain practical limitations. Multispectral data were collected using a DJI Mavic 3 UAV at a flight altitude of 30 m. Although this platform offers advantages of flexibility, efficiency, and high spatial resolution, it still presents limitations in practical applications. The multispectral sensor onboard the UAV has a limited number of bands and low spectral resolution, making it difficult to capture the fine spectral characteristics of crops and potentially affecting the inversion accuracy of some weak physiological and biochemical indicators. In addition, low-altitude flight is susceptible to external interference such as illumination, atmosphere, wind speed, and flight attitude, which reduces the stability and consistency of the data. Moreover, UAV-based multispectral remote sensing is sensitive to meteorological conditions; overcast, cloudy, or uneven illumination can lead to distortion of spectral reflectivity and a decrease in signal-to-noise ratio. For this reason, data in this study were only collected under clear and stable illumination conditions. In regions with frequent rainy seasons and cloudy skies, relying solely on this method would significantly increase the difficulty and cost of data acquisition. Furthermore, constrained by experimental conditions and observation objectives, this study only conducted data acquisition and model construction at a flight altitude of 30 m, without validating the model performance at higher altitudes. Future research can expand the scope of flight altitudes and observation scales to analyze the impact of altitude on spectral information and model accuracy. Meanwhile, for scenarios with complex meteorological conditions, satellite remote sensing, ground real-time monitoring equipment, or multi-source data fusion technology can be integrated to establish more stable monitoring schemes and enhance the applicability and robustness of the model across different regions and meteorological conditions.

## 5 Conclusions

To address the problems of insufficient mining of temporal features, difficulty in separating image details from noise, and inadequate response to yield-sensitive features in current UAV-based multispectral maize yield prediction, which lead to substandard prediction accuracy, this study designed the WaveST-Yield spatio-temporal deep learning model based on 3D-CNN. The core modules of the model are as follows: SPE, designed based on ConvLSTM as the front end, captures the temporal dynamics and spatio-temporal correlations of multispectral data during the entire maize growth period; MFSR, designed based on HWD, replaces the first-layer downsampling of traditional 3D-CNN to achieve image detail preservation and noise decoupling; AYSR, designed based on CBAM and embedded after feature extraction, enhances the extraction of yield-sensitive features and suppresses irrelevant interference. The model effectively solves the existing shortcomings and improves prediction accuracy and generalization ability.

A high-quality dataset was constructed using UAV multi-temporal multispectral data from two maize plots, where maize was greenhouse-raised as seedlings then transplanted rather than directly sown. A plot-scale data cube with four spectral bands and 15 vegetation indices supported model training and validation. Five-fold cross-validation reduced experimental contingency: Field 1 achieved R²=0.870, RMSE = 1.767 t/ha; Field 2, which was heterogeneous, achieved R²=0.775, RMSE = 1.695 t/ha, both achieving effective performance improvement and showing stable adaptability. WaveST-Yield outperformed traditional and single-structure deep learning models, providing an efficient solution for plot-scale yield prediction.

Although the WaveST-Yield model constructed in this study performed well in plot-scale maize yield prediction, it had certain limitations. The model’s prediction performance depends on the spatiotemporal resolution of remote sensing data; reduced resolution or insufficient temporal observations will impair feature ex traction. The lightweight architecture has insufficient dimension adaptation in multi-source data fusion, and experiments were only conducted on maize datasets. Future research will focus on improving the model’s adaptability to low-resolution data, expanding experimental areas to enhance cross-ecological generalization, exploring multi-source data fusion optimization, and extending the model to multiple crop types to improve its practical application value and universality.

The constructed plot-scale multi-temporal remote sensing data cube, multi-band-vegetation index fusion strategy, and lightweight model support small-sample agricultural remote sensing yield prediction. The WaveST-Yield model avoids complex manual feature engineering, directly realizes end-to-end yield estimation from original remote sensing images, and is applicable for farmland promotion, smart agriculture and rapid yield monitoring.

## Data Availability

The raw data supporting the conclusions of this article will be made available by the authors, without undue reservation.
